# Portraying Ethical Risks of Medical AI: Mixed Methods Study From Connotation Definition to a Survey on Physicians’ Cognition

**DOI:** 10.2196/89300

**Published:** 2026-07-09

**Authors:** Aiyi Zhang, Jing Yu, Qiaozhen Tao, Ting Zhou, Aijuan Sheng, Rui Li, Xiaojing Wu, Zhongguang Yu

**Affiliations:** 1 School of Population Medicine and Public Health, Peking Union Medical College & Chinese Academy of Medical Sciences, Beijing, China; 2 School of Management, Beijing University of Chinese Medicine, Beijing, China; 3 The First Affiliated Hospital, Jiangxi Medical College, Nanchang University, Nanchang, China; 4 General Practice, The First Affiliated Hospital of Chongqing Medical University, Chongqing, China; 5 Ethics Committee Office, Beijing You An Hospital, Capital Medical University, Beijing, China; 6 Respiratory Centre, China–Japan Friendship Hospital, No.2 Yinghua East Street, Beijing, Chaoyang District, 100029, China, 86 010-84026468

**Keywords:** medical artificial intelligence, ethical risks, connotation definition, physician cognition, influencing factors

## Abstract

**Background:**

Ethical risks of medical artificial intelligence (AI) are a global concern, but existing understanding remains fragmented without an integrated framework, and physicians’ awareness of these ethical risks is unclear.

**Objective:**

This study aimed to construct a multidimensional ethical risk framework for medical AI in the Chinese context, assess physicians’ perceptions of these risks, and provide theoretical support for AI risk governance.

**Methods:**

In the first phase, we conducted semistructured interviews with 36 experts (102,000-word transcript), analyzed via grounded theory (NVivo 11 [Lumivero]), yielding 5 main risk categories and 15 subcategories. In the second phase, a 21-item questionnaire based on this framework was administered to 600 physicians across 19 Chinese provinces. After the reliability and validity of the questionnaire, descriptive statistics, and multiple linear regression identified risk perceptions and influencing factors.

**Results:**

The framework includes physiological risks (eg, diagnostic error and improper treatment), psychological risks (eg, physicians’ technical anxiety and patient’s psychological anxiety), data and privacy risks (eg, privacy leakage and data security), social risks (eg, trust crisis, occupational impact, unclear liability, and autonomy erosion), and economic and sustainability risks (eg, increased financial burden, resource waste, environmental pollution, and energy consumption). Physicians (n=600) showed the highest concern for data and privacy risks, ambiguous accountability, and a physician-patient trust crisis. Economic and sustainability risks received the lowest agreement. Multiple linear regression identified significant predictors for risk perception. Specialized AI training was positively associated with perceptions of misdiagnosis risks (*β*=0.230, 95% CI 0.031‐0.429; *P*=.02), privacy leaks (*β*=0.220, 95% CI 0.041‐0.399; *P*=.02), and unclear liability (*β*=0.285, 95% CI 0.110‐0.460; *P*=.002). The establishment of medical institution AI ethics review procedures was positively associated with perceptions of diagnostic errors (*β*=0.355, 95% CI 0.141‐0.569; *P*=.001) and unclear liability (*β*=0.390, 95% CI 0.200‐0.580; *P*<.001), while AI unfamiliarity was negatively associated with trust crisis (*β*=−0.260, 95% CI −0.450 to −0.070, *P*=.006).

**Conclusions:**

This study proposes a contextualized ethical risk framework for medical AI in China to guide targeted governance. It is recommended that future efforts should focus on enhancing the ethical training of medical professionals, improving the ethical review mechanisms for AI in health care institutions, and clarifying the division of liabilities and accountability. These measures will promote the robust development of medical AI within an ethically compliant framework.

## Introduction

In the current era of in-depth integration of digitalization and intellectualization, artificial intelligence (AI) technology is reshaping the health care ecosystem at an unprecedented speed [1]. From AI-assisted intelligent medical image interpretation to intelligent auxiliary decision-making for specific clinical diseases, the application scenarios of AI in health care are constantly expanding, offering new opportunities to enhance medical efficiency and improve the accessibility of health care services [2,3]. However, with the widespread penetration of AI technology, the potential ethical risks behind it have gradually emerged, attracting great attention from the academic community and all sectors of society. According to the statistics and monitoring of the AI Incidents Monitor by the Organization for Economic Co-operation and Development, a total of 6926 AI-related risk incidents occurred globally in 2024, among which 360 were health care–related [4]. These risk incidents mainly stem from algorithmic bias, privacy and security breaches, liability and rights disputes, and technology misuse. Without ethical constraints, the abuse of AI technology may lead to severe negative social impacts, deviating from its original purpose of improving medical services and enhancing the efficiency of diagnosis and treatment [5,6]. Recognizing the severity of ethical issues, countries around the world have successively introduced relevant policies and guiding documents. For instance, the World Health Organization (WHO) put forward 6 ethical principles for AI in health in its *Ethics and Governance of Artificial Intelligence for Health* [7]; the European Union’s *Artificial Intelligence Act* imposes extremely high requirements on the training datasets of medical AI systems [8]; and the Canadian Association of Radiologists has released a white paper, providing a framework for the study of legal and ethical issues of AI in the field of medical imaging [9]. Other countries, including China and the United States, have also released their own guidance documents on the application of medical AI [10,11]. In recent years, the academic community has been continuously deepening the discussion of the core ethical issues of medical AI, and promoting the extension of relevant principles to an operable and assessable governance framework. Research indicates that the transparency and interpretability of algorithms are not only technical requirements but also the cornerstone for maintaining doctor-patient trust and clinical safety. The lack of an understandable decision-making logic can undermine doctors’ professional judgment, increase the uncertainty of the diagnosis and treatment process, and may compromise patient safety [12]. In terms of liability definition, scholars have called for the establishment of a systematic accountability framework to clarify the joint and differentiated responsibilities of developers, medical institutions, and clinicians in AI-assisted decision-making errors [13]. In addition, an increasing number of studies emphasize that the concerns of AI ethics must go beyond the technical effectiveness itself and should be deeply integrated with the core values of safeguarding patients’ well-being, dignity, and rights. This includes building trust by enhancing system transparency, protecting patients’ data privacy and autonomy, and eliminating biases originating from data or model design, so as to implement the people-centered ethical purpose in the application of technology [14,15]. The introduction of these policies, guidelines, and academic research has established robust institutional support for the healthy and orderly development of AI technology in the global medical field, steering its application toward a direction that aligns with ethics and safeguards public interest.

A review of the literature reveals that the ethical risks in the application of medical AI are primarily manifested in the following aspects: first, data security and privacy risks: AI systems rely on massive amounts of sensitive medical data for training and optimization, including private information, such as patient medical history, laboratory reports, and medical image records. Once vulnerabilities emerge in data security protections, data breaches can seriously threaten patient privacy and information security [16,17]. Second, insufficient algorithm transparency and explainability: the “black box” nature of medical AI algorithms poses significant challenges; although AI demonstrates high accuracy and efficiency in tasks, such as disease diagnosis and treatment recommendation, its decision-making process remains obscure and difficult to interpret clearly to physicians and patients. In complex and difficult cases, physicians are unable to effectively trace the reasoning behind AI decisions, which may increase the risk of misdiagnosis or missed diagnosis, hinder effective physician-patient communication and trust, and even lead to medical disputes and legal controversies [18]. Third, ambiguous accountability: this represents a thorny issue in the development of medical AI; when AI participates in clinical decision-making and leads to adverse outcomes, there is currently no clear and consistent legal framework or standard to specify whether primary responsibility should fall on the model developers, medical institutions, or the physicians using the AI [19,20]. This not only complicates the attribution of liability in medical accidents but may also prevent patients from receiving effective protection and fair compensation when their rights are infringed. The nature of ethical risks in medical AI is multidimensional and cross-level, encompassing issues such as data security, algorithmic fairness, accountability, transparency, and humanistic care [21-23].

However, existing research on these ethical risks often remains confined to single-dimensional analyses or fragmented discussions, lacking a systematic framework or comprehensive integration that fully captures the holistic landscape and intrinsic connections of ethical risks in medical AI. Moreover, as core participants in clinical practice, physicians are not merely users of medical AI tools, but also the “first line of defense” in identifying ethical risks. In the current context, where AI is deeply integrated into the diagnostic and treatment process, from verifying the rationality of AI-assisted diagnostic results to translating AI decision-making logic into layman’s terms for patients and detecting potential risks, such as data privacy breaches and algorithmic bias, physicians’ proactive identification and intervention are critical in every key link of clinical application. Their competence in AI and sensitivity to ethical risks directly determine their ability to identify risks. A lack of systematic understanding of AI may not only delay risk warnings but also undermine physician-patient trust because of the inability to explain the logic of AI applications to patients. Therefore, a deep understanding among physicians of the ethical risks associated with medical AI is not only a prerequisite for the sustainable application of AI but also the foundation of safeguarding patient rights and a core support for the implementation of industry-wide ethical standards.

Based on this, this study is grounded in the Chinese medical practice context and aims to systematically answer the following four core questions: (1) What is the specific connotation of medical AI ethics in the Chinese medical practice context? (2) How should the systematic core dimensions of medical AI ethics be summarized and defined? (3) From the perspective of physicians, what is the current state of physicians’ cognition regarding each specific dimension of medical AI ethics? (4) What key factors influence physicians’ level of cognition regarding medical AI ethics? To address these questions, this study first constructs a multidimensional ethical risk framework for medical AI using grounded theory analysis of semistructured expert interviews; subsequently, it assesses physicians’ perceptions of these ethical risks through a survey and identifies key influencing factors.

This study will further advance research on medical AI ethics. Theoretically, it establishes a localized multidimensional ethical risk framework for medical AI, filling gaps in systematic research, clarifying physicians’ ethical risk perception characteristics and influencing factors, and enriching theoretical achievements in this field. Practically, this study identifies physicians’ concerns regarding AI ethical risks. It provides empirical evidence for medical institutions to optimize AI ethical review mechanisms and deliver ethics training for physicians, so as to promote the sound and sustainable development of medical AI.

## Methods

### Overview

This study was conducted in China and consists of 2 parts. The first part used a grounded theory approach to analyze and construct an ethical risk framework for the clinical application of medical AI [24]. The second part involved the preliminary design of a questionnaire based on this framework, which is then revised and refined through focus group discussions [25], forming the final survey questionnaire to conduct the subsequent survey [26].

### Theoretical Framework

#### Research Subject

This study adopted a semistructured interview approach to gather experts’ authentic descriptions and perspectives on the influencing mechanisms and types of ethical risks associated with medical AI. We included a total of 36 experts, including 20 clinical experts, 11 ethics experts, 3 digital information experts, and 2 legal experts. We conducted the interviews between June and July 2024 and, with participants’ consent, audio-recorded them. The interview questions were as follows: (1) From an ethical standpoint, what risks do you believe AI poses in medical applications? (2) What influencing factors do you think contribute to the occurrence of these risks? (3) What measures do you consider effective in mitigating these risks?

#### Research Process

##### Overview

We transcribed interviews with 36 experts into electronic text, forming a systematically categorized raw corpus of 102,000 words. Among the 36 experts included in the analysis, textual data from 30 experts were used for formal analysis, while data from the remaining 6 experts were reserved for testing theoretical saturation. The research strictly followed the modeling guidelines and procedures of grounded theory. NVivo 11 software (Lumivero) was used to store, organize, code, and analyze the textual materials. To ensure trustworthiness, we adhered to the criteria by Braun and Clarke [27]. Credibility was achieved through 3 independent coders (AZ, YJ, and ZY) from clinical, ethical, and digital health backgrounds. Dependability was ensured by maintaining a full audit trail (transcripts, coding logs, NVivo files, and memos). Confirmability involved peer debriefing with 2 external qualitative methodologists and explicit documentation of preconceptions. For transferability, we provide a thick description of the study context and participant characteristics. In addition to the criteria above, we specifically addressed coder disagreement and reliability as detailed in the preceding paragraph, further strengthening the dependability and confirmability of our qualitative analysis. The specific steps are further described in the study.

##### Step 1: Open Coding

Open coding followed a raw data-labeling-conceptualization-categorization. From the interviews, we obtained 536 raw data segments reflecting ethical risks of medical AI. With reference to the 6 ethical principles proposed in the *Ethical Governance of Artificial Intelligence in Health Care guideline issued* by the WHO, initial concepts were refined iteratively, and ultimately 15 initial concepts were developed, such as diagnostic errors, improper treatment, patients’ psychological anxiety, physicians’ technical anxiety, data security, privacy leakage, weakened autonomy, unclear liability, fairness concerns, occupational impact, trust crisis, increased financial burden, resource waste, energy consumption, and environmental pollution.

##### Step 2: Axial Coding

Based on open coding, axial coding reintegrates dispersed concepts into a more structured theoretical framework. Through axial coding, this study categorizes the interrelationships and logical sequences among different categories at the conceptual level. A total of 6 main categories were identified, including physiological risks, psychological risks, economic risks, data and privacy risks, social risks, and environmental risks. To ensure transparency in our qualitative process, the following measures were adopted: (1) code merging—after independent open coding, the 3 coders (AZ, YJ, and ZY) met weekly to group similar concepts into categories based on semantic and conceptual agreement, requiring at least 2 coders’ consensus for each grouping; and (2) disagreement handling—coding disagreements accounting for approximately 10% were resolved through group discussions, and unresolved disputes were finally judged by a senior researcher (ZY).

##### Step 3: Selective Coding

Based on the results of open coding and axial coding, this study distilled the core category: “types of ethical risks in medical AI.” The questionnaire content was systematically optimized and refined, ultimately forming a medical AI risk perception framework encompassing physiological risks, psychological risks, data and privacy risks, social risks, and economic and sustainability risks.

##### Step 4: Theoretical Saturation Test

To ensure the reliability of coding, this paper reserved 6 sets of data in advance for the test of theoretical saturation and conducted the same coding analysis procedure. The analysis was cross-checked with the previously derived concepts, categories, and relationships between categories. The examination revealed no new categories or relationships, and no substantial disagreements were found in the existing categories and their relationships. Therefore, the theoretical saturation has passed the test.

### Questionnaire Survey

#### Questionnaire Design

Based on the grounded theory–derived framework of risk perception in medical AI, this study assembled a focus group of 11 experts specializing in clinical medicine, AI, and medical ethics. Using the focus group discussion method, the preliminary questionnaire framework underwent multiple rounds of deliberation and revision. To ensure a transparent and replicable translation of the qualitative framework into a quantitative instrument, we followed a systematic, multistep process. First, the research team mapped the 15 subcategories derived from open coding (eg, B1 “diagnostic errors,” and B5 “data security”) directly onto preliminary questionnaire items. Multifaceted subcategories were split into multiple items (eg, B3 “physicians’ technical anxiety” was split into Q3 and Q4), while highly related subcategories were merged after group discussion. The first round focused on the completeness of questionnaire dimensions and the clarity of item wording. Clinical experts supplemented practical items such as “disease surveillance and early warning,” while AI experts refined the technical accuracy of terms like “algorithmic black box,” resulting in a revised version containing 26 items. The second round involved in-depth discussions on the scale’s applicability, leading to the deletion of 2 duplicate items and the merging of 3 redundant items, ultimately forming a final questionnaire comprising 21 items. The questionnaire consists of 2 parts: the first collects demographic information of the experts, including gender, educational background, professional title, and years of work experience; and the second investigates physicians’ perceptions of ethical risks in the clinical application of medical AI, using a 5-point Likert scale ranging from “strongly disagree” to “strongly agree.” The reporting adhered to the COREQ (Consolidated Criteria for Reporting Qualitative Research) checklist (Checklist 1). For the quantitative component, we also followed the STROBE (Strengthening the Reporting of Observational Studies in Epidemiology) checklist for cross-sectional studies (Checklist 2). A detailed mapping table linking qualitative codes, risk dimensions, and final questionnaire items is provided in Multimedia Appendix 1.

#### Questionnaire Survey and Implementation

This cross-sectional survey was conducted between June and August 2024 across 19 provinces in China, encompassing tertiary, secondary, and primary hospitals to ensure institutional diversity. Eligible participants were practicing physicians with at least 1 year of clinical experience who provided informed consent and voluntarily agreed to complete the questionnaire. No restrictions were placed on specialty, age, or gender.

A 4-step systematic recruitment strategy was implemented to maximize reach while minimizing duplication and selection bias. First, relying on the internal communication system of cooperative medical institutions, we distributed an electronic questionnaire link along with a brief study information sheet to all eligible physicians. Second, to capture physicians from institutions without formal collaboration, we posted recruitment information in verified WeChat (Tencent Holdings Limited) groups of provincial medical associations (eg, Beijing, Jiangxi, and Chongqing, as representative examples). Third, we encouraged physicians who had participated in the survey to invite and share the questionnaire with their eligible peers. Through this method, the final sample comprised physicians from 19 provinces in China, including Beijing, Guangdong, Zhejiang, Jiangsu, Sichuan, Hubei, Shaanxi, and others. To avoid duplicate responses, each physician was assigned a unique anonymous ID generated by the survey platform based on their mobile phone number. Finally, before the formal investigation, 5 representative samples were invited to participate in a pretest to structurally assess the clarity and logical coherence of the items. Feedback was used to optimize the scale framework.

In this round of the survey, a total of 619 questionnaires were collected. Invalid responses (eg, incomplete answers, duplicate submissions, or illogical response patterns) were excluded. After screening, resulting in 600 valid samples were retained, resulting in an effective response rate of 96.93%. Reliability and validity tests indicated that the overall Cronbach α of the scale exceeded the acceptable threshold of 0.7, demonstrating satisfactory internal consistency. To assess structural validity, we conducted confirmatory factor analysis rather than exploratory factor analysis, given that our study had a prespecified theoretical framework comprising 5 distinct dimensions derived from grounded theory. The purpose of this confirmatory factor analysis was to test whether the empirical data fit this hypothesized multidimensional structure. All 21 items demonstrated standardized factor loadings greater than 0.6 on their respective intended dimensions, confirming strong structural validity and supporting the construct validity of our 5D framework.

### Statistical Analysis

A multiple linear regression model was used to identify key predictors of risk perception. Independent variables were selected based on literature review and clinical relevance, including years of service, hospital level, familiarity with AI, training experience, and the establishment of medical institution AI ethics review procedures. For categorical predictors, the following reference categories were used: years of service (>15 y), hospital level (secondary and primary hospitals), familiarity with AI (familiar), training experience (none), and establishment of AI ethics review procedures (no). Significant variables were incorporated into the model, and results were detailed with β coefficients, 95% CIs, and *P* values. All analyses were performed using IBM SPSS Statistics 26.

### Ethical Considerations

All participants provided electronic informed consent before the study began. This study involves an anonymous online questionnaire approved by the Ethics Committee of China-Japan Friendship Hospital (approval 2024-KY-254). Instead of providing physical signatures, participants reviewed an informed consent statement on the survey’s opening page. This statement detailed the study’s purpose, its voluntary nature, confidentiality protocols, and the right to withdraw. Respondents had to click “I have read and agree to participate” to access the questionnaire. No compensation was offered to participants, as the survey was voluntary, posed minimal risk, and placed no financial burden on respondents. Data collected were anonymized, encrypted, and strictly limited to academic research purposes.

## Results

### Ethical Risk Identification Framework for Medical AI

Based on the grounded theory approach, this study ultimately distilled a theoretical framework with “types of ethical risks in medical AI” as the core category. This framework covers 5 main categories, namely physiological risks, psychological risks, data and privacy risks, social risks, and economic and sustainability risks, along with their 15 subcategories (Table 1). Specifically, physiological risks refer to potential or actual physical harm to patients resulting from misdiagnosis or mistreatment due to algorithmic biases, limited data samples, or technical malfunctions in the process of AI-assisted diagnosis and treatment [28]. Psychological risks involve patients’ distrust and anxiety toward AI-driven decisions, as well as technical anxiety among health care providers caused by overreliance on AI [29,30]. Data and privacy risks denote security issues such as the leakage, misuse, or unauthorized secondary use of patient private data during data collection, storage, transmission, and usage by medical AI systems [31,32]. Social risks encompass multidimensional socioethical issues arising from the application of medical AI, including equity concerns stemming from uneven allocation of AI resources across regions [33]; the physician-patient trust crisis caused by the opacity of AI decisions, and the weakened decision-making autonomy of both physicians and patients due to the intervention of AI technology [34]; ambiguity in liability attribution among developers, medical institutions, and users when medical errors occur [35]; and occupational impacts caused by AI-driven substitution pressure on physicians’ roles [36]. Economic and sustainability risks mainly refer to the financial burden placed on medical institutions due to high costs related to the development, procurement, and maintenance of medical AI, as well as sustainability challenges, such as excessive energy consumption, resource waste, and environmental pollution resulting from rapid technological iteration [37].

**Table 1.** Ethical risk framework for medical artificial intelligence.

Encoding, main category, and serial number	Concrete category
C1: physiological risks	
B1	Diagnostic error
B2	Improper treatment
C2: psychological risks	
B3	Physicians’ technical anxiety
B4	Patient’s psychological anxiety
C3: data and privacy risks	
B5	Data security
B6	Privacy leak
C4: social risks	
B7	Fairness concerns
B8	Occupational impact
B9	Trust crisis
B10	Unclear liability
B11	Autonomy erosion
C5: economic and sustainability risks	
B12	Increased financial burden
B13	Resource waste
B14	Environmental pollution
B15	Energy consumption

### Basic Respondent Demographic Statistics

This study included 600 physicians, and 0 people refused to participate. The characteristics of the sample group are as presented in Table 2. In terms of sex, males accounted for 46% (n=276) and females accounted for 54% (n=324); regarding age groups, physicians aged 40 years and younger accounted for 51% (n=306), those aged 41‐50 years accounted for 34.5% (n=207), while physicians aged 51 years and older accounted for only 14.5% (n=87). The majority worked in tertiary hospitals (n=375, 62.5%), while 37.5% (n=225) were affiliated with secondary and primary hospitals. Physicians with a bachelor’s degree or below accounted for 61% (n=366), and those with a postgraduate degree accounted for 39% (n=234). Regarding professional titles, resident physicians account for 14.5% (n=87), attending physicians account for 39.8% (n=239), associate chief physicians account for 30.8% (n=185), and chief physicians account for 14.8% (n=89). In terms of years of practice, physicians with 15 years or less of practice accounted for 54.3% (n=326), and those with more than 15 years of practice accounted for 45.7% (n=274). Most physicians reported a moderate to low level of familiarity with medical AI, 21.7% (n=130) indicated they were familiar, 47.7% (n=286) were neutral, and 30.7% (n=184) were unfamiliar. Additionally, 21% (n=126) had received specialized AI training, 33.8% (n=203) had self-studied topics related to medical AI, and 45.2% (n=271) had received no training at all. In clinical practice, 55.2% (n=331) had used AI, while 44.8% (n=269) had not. Furthermore, the development of ethical governance systems appears significantly underdeveloped; only 16.8% (n=101) reported that their hospital had established standard ethical review procedures for medical AI, 37.5% (n=225) stated that no such procedures were in place, and 45.7% (n=274) were unaware of whether such procedures existed.

**Table 2.** Respondent Information.

Characteristic	Response, n (%)
Sex	
Male	276 (46)
Female	324 (54)
Age group (y)	
≤40	306 (51)
41‐50	207 (34.5)
≥51	87 (14.5)
Hospital level	
Tertiary hospital	375 (62.5)
Secondary and primary hospital	225 (37.5)
Educational level	
Less than a Bachelor of Medicine	32 (5.3)
Bachelor of Medicine	334 (55.7)
Higher than a bachelor’s degree	234 (39)
Professional title	
Resident physician	87 (14.5)
Attending physician	239 (39.8)
Associate chief physician	185 (30.8)
Chief physician	89 (14.8)
Years of service	
≤15	326 (54.3)
>15	274 (45.7)
Are you familiar with the application of medical artificial intelligence in clinical work?	
Unfamiliar	184 (30.7)
Neutral	286 (47.7)
Familiar	130 (21.7)
Have you received specialized training in AI^a^?	
Specialized training	126 (21)
Self-study	203 (33.8)
None	271 (45.2)
Have you ever used AI in your clinical diagnosis and treatment process?	
Yes	331 (55.2)
No	269 (44.8)
Has the hospital established an ethical review process for AI technology?	
Yes	101 (16.8)
No	225 (37.5)
Unclear	274 (45.7)

a AI: artificial intelligence.

### Physicians’ Awareness of Ethical Risks in the Clinical Application of Medical AI

**Figure 1.**
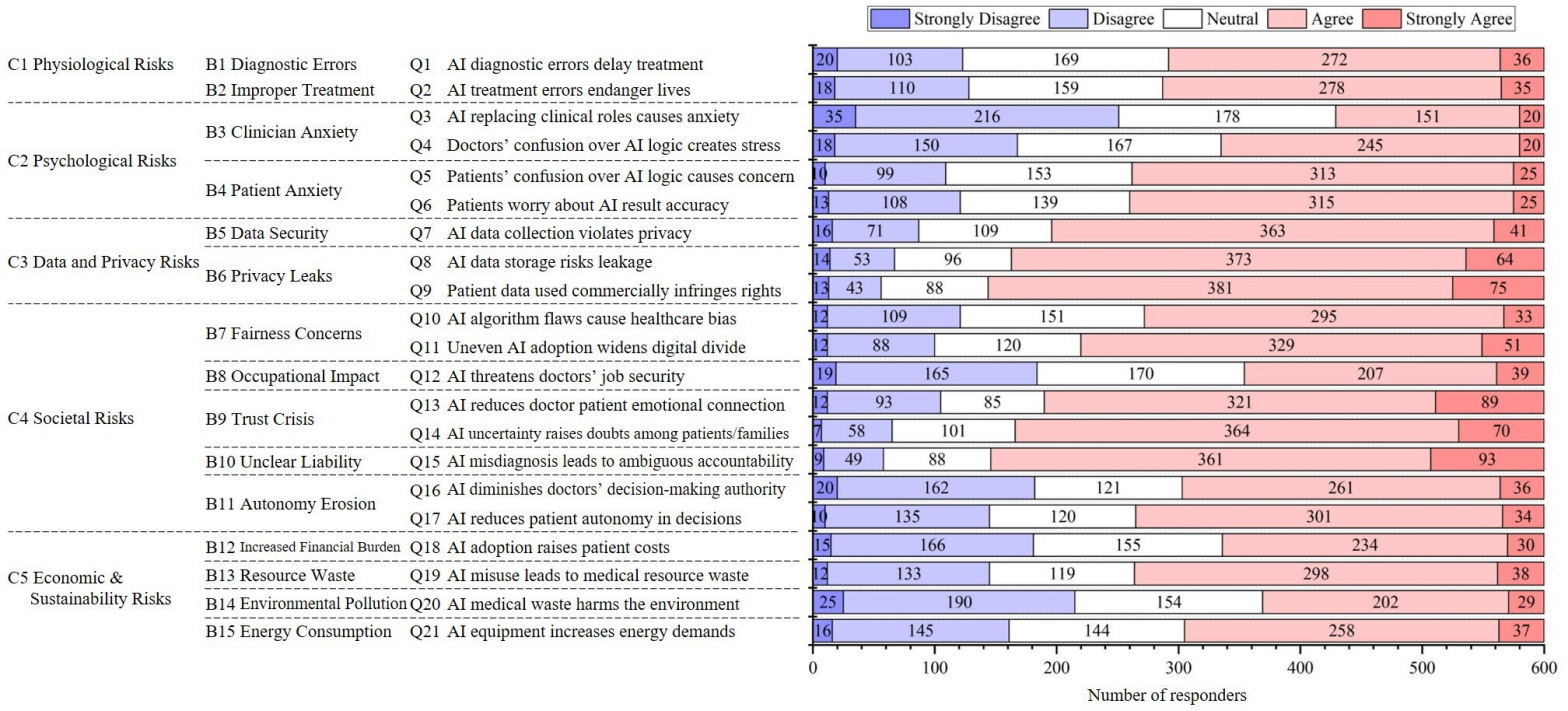
Physicians’ perception of ethical risks in medical artificial intelligence (this is a bar chart visually summarizing the agreement percentages for key items across the five risk dimensions described above). AI: artificial intelligence.

This study used a structured questionnaire survey to systematically analyze physicians’ perceptions of risks in medical AI applications across 5 dimensions—physiological risks, psychological risks, data and privacy risks, social risks, and economic and sustainability risks (Figure 1).

Regarding the perception of physiological risks (C1), for Q1 “AI diagnostic errors delay treatment,” 51.3% (n=308) of physicians expressed agreement, while only 20.5% (n=123) disagreed. For Q2 “AI treatment errors endanger lives,” 52.2% (n=313) of physicians indicated apprehension, 21.3% (n=128) did not believe that AI would pose such risks, and nearly one-third remained neutral.

Perceptions of psychological risks (C2) revealed a notable asymmetry between “patient psychological anxiety” and “physicians’ technical anxiety.” Regarding Q3 (“AI replacing clinical roles causes anxiety”), 28.5% (n=171) of physicians expressed agreement, while 41.8% (n=251) disagreed and 29.7% (n=178) remained neutral. As for Q4 (“doctors’ confusion over AI logic creates stress”), 44.2% (n=265) of physicians acknowledged such risk, whereas 55.5% (n=335) denied it. In contrast, issues related to patient psychological anxiety received broader consensus. Specifically, 56.7% (n=338) of physicians agreed with Q5 (“Patients’ confusion over AI logic causes concern”), and an equal proportion of 56.7% (n=340) endorsed Q6 (“Patients worry about AI result accuracy”).

Data and privacy risks (C3) emerged as the dimension with the highest consensus among physicians. A majority expressed agreement with Q7 (“AI data collection violates privacy”; n=404, 67.3%), Q8 (“AI data storage risks leakage”; n=437, 72.8%), and Q9 (“Patient data used commercially infringes rights”; n=456, 76%). Neutral attitudes were reported by 18.2% (n=109), 16% (n=96), and 14.7% (n=88) of respondents, respectively, while disagreement was less than 14.5% (87/600) across all items. These results indicate a high level of shared concern regarding such risks among the physician community.

Regarding social risks (C4), Q15 (“AI misdiagnosis leads to ambiguous accountability”) received the highest agreement rate at 75.7% (n=454), with only 9.7% (n=58) expressing disagreement. Physician-patient trust crisis also drew significant attention, with agreement rates of 68.3% (n=419) for Q13 (“AI reduces physicians’ patient emotional connection”) and 72.3% (n=434) for Q14 (“AI uncertainty raises doubts among patients/families”). Notable divergence was observed concerning Q12 (“AI threatens physicians’ job security”), with 41% (n=246) in agreement and 30.7% (n=184) in disagreement, while 28.3% (n=170) remained neutral. Perceptions of fairness risks revealed a mix of concern and skepticism. The agreement rates for Q10 (“AI algorithm flaws cause healthcare bias”) and Q11 (“uneven AI adoption widens digital divide”) were 54.7% (n=328) and 63.3% (n=380), respectively. Most physicians agreed with autonomy erosion (Q16 and Q17), with agreement rates of 49.5% (n=297) and 55.8% (n=335), respectively.

Perceptions of economy and sustainability risks (C5) were the lowest among all dimensions. Only Q19 “AI misuse leads to medical resource waste” received agreement from over half of the respondents (n=336, 56%). The other issues have not yet garnered widespread attention among physicians; Q18 “AI adoption raises patient costs,” Q20 “AI medical waste harms the environment,” and Q21 “AI equipment increases energy demands” received agreement rates of merely 44% (n=264), 38.5% (n=231), and 49.2% (n=295), respectively.

### Analysis of Differences in Physicians’ Perception of Ethical Risks in Medical AI

This study used a multiple regression model to analyze the effects of years of practice, familiarity with AI, training experience, hospital level, and the establishment of standard AI ethics review procedures on physicians’ risk perceptions across the 5 major dimensions—physiological, psychological, data and privacy, social, and economic and sustainability risks. The key findings reveal a heterogeneous nature of risk perceptions across different dimensions, with each factor demonstrating varying associations with such perceptions (Table 3).

Multiple linear regression analysis revealed that perceptions of physiological risks were significantly influenced by the establishment of standard medical AI ethics review procedures, years of work experience, familiarity with AI, and training experience, among which the implementation of ethics review protocols demonstrated the most substantial effect. Specifically, working in hospitals with established ethics review procedures was significantly positively associated with physicians’ perception of both misdiagnosis (*β*=0.355, *P*=.001) and mistreatment risks (*β*=0.355, *P*=.001). Compared with highly experienced physicians, those with no more than 15 years of practice reported higher perception of misdiagnosis risks (*β*=2.712, *P*=.02). Physicians unfamiliar with medical AI demonstrated lower perception of misdiagnosis risks than their familiar counterparts (*β*=–0.235, *P*=.03). Furthermore, Physicians who had received specialized training showed a significantly more positive perception of misdiagnosis risks compared with those without any training (*β*=0.230, *P*=.02).

Regarding the dimension of psychological risk perception, only familiarity with AI demonstrated a statistically significant influence. Other factors may affect the perception of psychological risks through indirect pathways, although their effects are relatively weak and insufficient to achieve statistical significance. Specifically, compared with physicians familiar with AI, those who were very unfamiliar showed a negative correlation in their perception of both technical anxiety among physicians (*β*=–0.300, *P*=.007) and psychological anxiety among patients (*β*=–0.295, *P*=.006). Similarly, physicians with moderate familiarity also demonstrated a negative correlation in technical anxiety among physicians (*β*=–0.230, *P*=.02) and psychological anxiety among patients (*β*=–0.205, *P*=.04) compared with those familiar with AI.

In the dimension of data and privacy risk perception, the establishment of medical AI ethics review procedures and training experience demonstrated statistically significant positive effects. Specifically, physicians working in hospitals with established AI ethics review protocols showed higher perception of both data security risks (*β*=0.230, *P*=.03) and privacy leakage risks (*β*=0.245, *P*=.01) compared with those in institutions without such procedures. Furthermore, individuals who had received specialized training displayed a more acute perception of data and privacy risks than those without training (*β*=0.220, *P*=.02).

**Table 3.** Differences in physicians’ perception of ethical risks in medical AI^a^.

Variable	Years of service		Hospital level		Familiarity level			Training status			Establishment of AI ethics review standards		
	≤15	>15	Tertiary hospital	Secondary and primary hospital	Unfamiliar	Neutral	Familiar	Specialized training	Self-study	None	Yes	No	Unclear
B1. Diagnostic errors													
β (95% CI)	2.712 (0.486 to 4.938)	ref^b^	0.631 (–0.784 to 2.046)	ref	–0.235 (–0.448 to –0.022)	–0.165 (–0.364 to 0.034)	ref	0.230 (0.031 to 0.429)	0.090 (–0.082 to 0.262)	ref	0.355 (0.141 to 0.569)	0.115 (–0.053 to 0.283)	ref
*P* value	.02	ref	.39	ref	.03	.097	ref	.02	.31	ref	.001	.18	ref
B2. Improper treatment													
β (95% CI)	2.239 (–0.054 to 4.532)	ref	0.223 (–1.204 to 1.649)	ref	–0.21 (–0.420 to 0.000)	–0.155 (–0.350 to 0.040)	ref	0.14 (–0.060 to 0.340)	0.085 (–0.090 to 0.260)	ref	0.355 (0.141 to 0.569)	0.185 (0.022 to 0.348)	ref
*P* value	.06	ref	.76	ref	.06	.12	ref	.17	.33	ref	.001	.03	ref
B3. Physicians’ technical anxiety													
β (95% CI)	1.398 (–0.675 to 3.470)	ref	0.233 (–1.184 to 1.650)	ref	–0.300 (–0.518 to –0.082)	–0.230 (–0.429 to –0.031)	ref	0.125 (–0.080 to 0.330)	0.100 (–0.080 to 0.280)	ref	–0.085 (–0.310 to 0.140)	–0.085 (–0.260 to 0.090)	ref
*P* value	.19	ref	.75	ref	.007	.02	ref	.25	.28	ref	.45	.34	ref
B4. Patient’s psychological anxiety													
β (95% CI)	2.189 (–0.257 to 4.636)	ref	–1.478 (–3.147 to 0.186)	ref	–0.295 (–0.509 to –0.081)	–0.205 (–0.400 to –0.010)	ref	0.130 (–0.070 to 0.330)	0.010 (–0.160 to 0.180)	ref	0.205 (–0.010 to 0.420)	0.065 (–0.100 to 0.230)	ref
*P* value	.08	ref	.08	ref	.006	.04	ref	.19	.92	ref	.06	.46	ref
B5. Data security													
β (95% CI)	2.150 (–0.207 to 4.508)	ref	–0.843 (–2.323 to 0.640)	ref	–0.120 (–0.320 to 0.080)	–0.020 (–0.200 to 0.160)	ref	0.175 (–0.010 to 0.360)	–0.090 (–0.250 to 0.070)	ref	0.230 (0.026 to 0.434)	0.045 (–0.110 to 0.200)	ref
*P* value	.07	ref	.27	ref	.22	.84	ref	.06	.25	ref	.03	.597	ref
B6. Privacy leaks													
β (95% CI)	0.854 (–1.839 to 3.549)	ref	–0.498 (–2.071 to 1.078)	ref	–0.15 (–0.340 to 0.040)	0.000 (–0.180 to 0.180)	ref	0.22 (0.041 to 0.399)	–0.07 (–0.230 to 0.090)	ref	0.245 (0.050 to 0.440)	0.03 (–0.120 to 0.180)	ref
*P* value	.54	ref	.54	ref	.13	≥.99	ref	.02	.38	ref	.01	.72	ref
B7. Fairness concerns													
β (95% CI)	2.347 (–0.989 to 5.684)	ref	–2.125 (–4.343 to 0.113)	ref	–0.175 (–0.380 to 0.030)	–0.060 (–0.250 to 0.130)	ref	0.220 (0.025 to 0.415)	0.015 (–0.150 to 0.180)	ref	0.150 (–0.060 to 0.360)	–0.08 (–0.240 to 0.080)	ref
*P* value	.17	ref	.06	ref	.09	.51	ref	.03	.85	ref	.16	.31	ref
B8. Occupational impact													
β (95% CI)	1.642 (–0.320 to 3.603)	ref	–1.743 (–3.216 to –0.270)	ref	–0.185 (–0.410 to 0.040)	–0.185 (–0.390 to 0.020)	ref	0.190 (–0.020 to 0.400)	0.120 (–0.060 to 0.300)	ref	0.045 (–0.180 to 0.270)	–0.025 (–0.200 to 0.150)	ref
*P* value	.10	ref	.02	ref	.11	.08	ref	.07	.197	ref	.69	.78	ref
B9. Trust crisis													
β (95% CI)	4.372 (1.152 to 7.592)	ref	–2.130 (–4.343 to 0.121)	ref	–0.260 (–0.450 to –0.070)	–0.145 (–0.320 to 0.030)	ref	0.265 (0.090 to 0.440)	0.110 (–0.040 to 0.260)	ref	0.330 (0.140 to 0.520)	0.035 (–0.110 to 0.180)	ref
*P* value	.008	ref	.06	ref	.006	.09	ref	.003	.17	ref	.001	.66	ref
B10. Unclear liability													
β (95% CI)	2.035 (–1.082 to 5.151)	ref	–1.345 (–3.219 to 0.529)	ref	0.155 (0.040 to 0.270)	–0.260 (–0.330 to 0.020)	ref	0.285 (0.110 to 0.460)	0.015 (–0.140 to 0.170)	ref	0.390 (0.200 to 0.580)	0.030 (–0.120 to 0.180)	ref
*P* value	.20	ref	.16	ref	.009	.08	ref	.002	.89	ref	<.001	.72	ref
B11. Autonomy erosion													
β (95% CI)	2.710 (–0.518 to 5.937)	ref	–0.949 (–3.147 to 1.257)	ref	–0.115 (–0.340 to 0.110)	–0.160 (–0.370 to 0.050)	ref	0.425 (0.210 to 0.640)	0.070 (–0.110 to 0.250)	ref	0.135 (–0.100 to 0.370)	–0.040 (–0.220 to 0.140)	ref
*P* value	.10	ref	.399	ref	.32	.14	ref	<.001	.45	ref	.25	.66	ref
B12. Increased financial burden													
β (95% CI)	1.102 (–1.363 to 3.568)	ref	1.04 (–0.620 to 2.700)	ref	–0.225 (–0.440 to –0.010)	–0.100 (–0.300 to 0.100)	ref	0.145 (–0.060 to 0.350)	0.135 (–0.040 to 0.310)	ref	0.080 (–0.140 to 0.300)	0.055 (–0.120 to 0.230)	ref
*P* value	.38	ref	.22	ref	.04	.33	ref	.17	.13	ref	.49	.53	ref
B13. Resource waste													
β (95% CI)	1.576 (–0.856 to 4.006)	ref	–0.176 (–1.754 to 1.404)	ref	–0.285 (–0.500 to –0.070)	–0.125 (–0.320 to 0.070)	ref	0.265 (0.060 to 0.470)	0.115 (–0.060 to 0.290)	ref	0.130 (–0.090 to 0.350)	0.090 (–0.080 to 0.260)	ref
*P* value	.20	ref	.83	ref	.01	.22	ref	.01	.19	ref	.25	.31	ref
B14. Environmental pollution													
β (95% CI)	–0.103 (–2.283 to 2.073)	ref	–0.096 (–1.492 to 1.300)	ref	–0.075 (–0.300 to 0.150)	0.075 (–0.130 to 0.280)	ref	0.195 (–0.020 to 0.410)	0.095 (–0.090 to 0.280)	ref	–0.13 (–0.360 to 0.100)	0.000 (–0.180 to 0.180)	ref
*P* value	.93	ref	.89	ref	.53	.49	ref	.07	.298	ref	.28	.98	ref
B15. Energy consumption													
β (95% CI)	0.279 (–1.814 to 2.375)	ref	0.412 (–1.085 to 1.908)	ref	–0.210 (–0.430 to 0.010)	–0.050 (–0.250 to 0.150)	ref	0.315 (0.110 to 0.520)	0.130 (–0.050 to 0.310)	ref	0.060 (–0.160 to 0.280)	–0.055 (–0.230 to 0.120)	ref
*P* value	.79	ref	.59	ref	.06	.62	ref	.003	.14	ref	.599	.54	ref

a AI: artificial intelligence.

b ref: reference.

In the dimension of social risk, hospital level, years of service, familiarity with AI, training experience, and the establishment of medical AI ethics review procedures demonstrated significant driving effects on risk perception, with training experience as the most influential factor. Specifically, physicians who had received specialized training showed higher perception than untrained counterparts in the following dimensions: fairness concerns (*β*=0.220, *P*=.03), trust crisis (*β*=0.265, *P*=.003), ambiguous accountability (*β*=0.285, *P*=.002), and diminished autonomy (*β*=0.425, *P*<.001). Compared with highly experienced physicians, those with no more than 15 years of service showed significantly higher perception of trust crisis (*β*=4.372, *P*=.008). Physicians unfamiliar with medical AI demonstrated significantly different perceptions in trust crisis (*β*=–0.260, *P*=.006) and ambiguous accountability (*β*=0.155, *P*=.009). The establishment of medical AI ethics review procedures was associated with significantly higher perception of both trust crisis (*β*=0.330, *P*=.001) and ambiguous accountability (*β*=0.390, *P*<.001) compared with institutions without such protocols. Notably, physicians in tertiary hospitals showed a negative effect in their perception of occupational impact compared with those in primary hospitals (*β*=–1.743, *P*=.02).

In the dimension of economic and sustainability risks, familiarity with AI and training status showed several notable associations. Specifically, physicians’ familiarity with AI showed a statistically significant negative correlation with perceptions of increased financial burden (*β*=–0.225, *P*=.04) and resource waste (*β*=–0.285, *P*=.01), and a negative correlation that did not reach statistical significance with energy consumption (*β*=–0.210, *P*=.06). Physicians who had received specialized training expressed significantly greater concern regarding resource waste (*β*=0.265, *P*=.01) and energy consumption (*β*=0.315, *P*=.003) compared with those without training.

## Discussion

### Principal Findings

This study achieved its two aims: (1) constructing a 5-dimensional ethical risk framework for medical AI, and (2) identifying physicians’ perceptions of these risks and identifying key influencing factors via a cross-sectional survey of 600 Chinese physicians. The constructed framework comprises 5 main categories and 15 subcategories. The survey revealed that physicians expressed the highest concern for data and privacy risks, ambiguous accountability, and a physician-patient trust crisis, while economic and sustainability risks received the lowest agreement. Multiple regression analysis further identified that specialized AI training, familiarity with AI, and the establishment of hospital AI ethics review procedures were significant predictors of risk perception.

### Establishing a Multidimensional Ethical Risk Framework for Medical AI

First, this study breaks through the limitations of a fragmented and isolated approach often found in existing research on medical AI risks, by constructing an ethical risk framework encompassing 5 main categories and 15 subcategories. While conceptual overlaps with existing international frameworks are inevitable given the universal nature of core ethical issues, the distinct contribution of our framework lies in (1) the explicit inclusion of economic and sustainability risks as a core ethical dimension; (2) its empirical derivation from Chinese clinical experts’ lived experiences rather than solely from normative analysis; and (3) its validation through physician cognitive surveys, which provides practical priorities for risk governance. This combined methodological and contextual novelty enhances the framework’s relevance for both local and global medical AI ethics discourse. Compared with current international guidelines, such as the WHO’s “*Ethics and Governance of Artificial Intelligence for Health*,” which focuses primarily on principled issues like data security, accountability, and fairness [38]; the European Union’s *Artificial Intelligence Act*, which emphasizes data governance, algorithmic transparency, human oversight, and particularly the explainability of high-risk medical AI systems and patient informed consent [39]; the US Food and Drug Administration (FDA) centers its review of AI medical devices on safety and effectiveness, but systematic ethical assessment has not yet become a mandatory requirement [40]; although relevant white papers in Canada propose professional ethical frameworks, they are mainly limited to single scenarios such as medical imaging and lack a holistic risk perspective across departments and multiple scenarios [41]. Starting from multiple dimensions including physical, psychological, data and privacy, social, and economic and sustainability aspects, this study incorporates numerous specific risks into the framework, such as diagnostic errors, improper treatment, data security, and privacy leakage. Furthermore, it innovatively includes economic and sustainability risks in ethical considerations, which aligns with the goals of “ensuring healthy lives” and “climate action” outlined in the United Nations Sustainable Development Goals [42]. This makes the discussion go beyond simple operations or environmental management. These issues raise fundamental ethical concerns, including equity in health care access, affordability for patients, and intergenerational justice, as rising operational costs may be passed on to users or exacerbate existing disparities in resource allocation across regions and institutions. A systemic, ethics-based examination of such impacts is essential to understand the long-term consequences of medical AI on the health care ecosystem as a whole. This systemic, ethics-based examination of long-term consequences provides a theoretical foundation for the ethical governance of medical AI in China and offers a Chinese approach to global governance.

### Physicians’ Awareness of Ethical Risks in Medical AI Drives Precise Governance of AI Risks

Our survey revealed that physicians’ ethical concerns are highly concentrated in 3 interconnected dimensions—data and privacy risks, ambiguous accountability, and physician-patient trust crisis. These findings provide evidence-based priorities for AI risk governance.

First, over 67% of physicians expressed strong concern about data security and privacy leakage, aligning with WHO guidelines. Both the European Union’s General Data Protection Regulation (GDPR) and the US HIPAA (Health Insurance Portability and Accountability Act) have established stringent frameworks for medical data protection, highlighting principles such as informed consent, data minimization, and restrictions on cross-border data transfer, thereby providing a legal foundation for data ethics in AI applications. However, practical challenges remain, including the secondary use of data and insufficient algorithmic transparency [43,44]. This high level of concern is particularly salient in the Chinese context; on the one hand, the Personal Information Protection Law is still in its early implementation stage, with 37.5% (225/600) of hospitals lacking standardized AI ethics review procedures; on the other hand, physician-patient relationships in China rely heavily on interpersonal trust, and data leakage risks directly threaten this foundation, thereby amplifying physicians’ professional anxiety. Since the implementation of *the Personal Information Protection Law of the People’s Republic of China*, significant legislative strengthening has occurred, although enforcement is still in its early stages. The fact that 37.5% (225/600) of hospitals have not yet established standardized AI ethics review procedures reveals gaps in the development of data governance mechanisms within medical institutions. Therefore, there is a practical need to refine detailed rules on data and patient privacy protection, prevent disorderly exploitation and misuse of data in sharing practices, ensure effective informed consent, and translate privacy principles into actionable operational protocols.

Second, 75.7% (454/600) of respondents identified ambiguous accountability as an urgent issue. The European Union’s *Artificial Intelligence Act* imposes strict regulations on medical AI systems, supplemented by *the AI Liability Directive*, which facilitates the determination of fault and causation [45]. In 2025, the European Parliament recommended a strict liability system for high-risk AI systems to ensure effective compensation for victims. The United States explores shared responsibility but lacks federal mandatory legislation. WHO guidelines call for human oversight to clarify responsibilities. However, China still lacks clear regulatory documents defining accountability. Moreover, data show that 45.7% (274/600) of physicians are unaware of hospital-level AI ethics procedures. This “fragmented ethical oversight” heightens concerns over uncertain accountability, especially among physicians with low AI literacy, who may avoid using AI for fear of assuming liability.

Third, about 70% (419/600) of physicians agreed that AI weakens emotional communication and threatens patient trust, reflecting disruption of the traditional physician-patient relationship, particularly in China, where interpersonal bonds are highly valued. WHO guidelines note that AI introduces a “physician-patient-AI” model, reducing time and opportunities for face-to-face communication and emotional empathy, which conflicts with China’s communication-centered medical culture. Additionally, WHO guidelines emphasize that medical AI is characterized by “technical black boxes” and “explanation black boxes.” The low transparency of algorithmic decision-making makes it difficult for patients to comprehend the logic behind AI-generated recommendations. This “uncertainty” can amplify patients’ skepticism toward diagnostic results, triggering a crisis of trust. The high level of concern among Chinese physicians regarding such risks stems from their acute awareness of the importance of “interpersonal bonds” and the “vulnerability of trust” within local physician-patient relationships. The trust crisis is influenced by specialized training, AI familiarity, and institutional ethics review standards. Therefore, AI ethics training and enhanced AI literacy are essential to rebuilding trust.

Finally, concerns about fairness and occupational impact varied significantly by hospital level. In our sample, 62.5% (375/600) of physicians worked in tertiary hospitals, and only 21% (126/600) had received specialized AI training, potentially underestimating the concerns of primary care physicians who face greater resource constraints. For instance, agreement rates for fairness (54.7%) and digital divide (63.3%) may be underestimated due to the limited representation of primary care physicians, who face greater resource constraints. Similarly, low concern about occupational impact likely reflects overrepresentation of tertiary hospital physicians, who feel less threatened by AI substitution. Thus, current findings may reflect a technology-advantaged perspective; actual risk perception may be higher, especially in resource-limited settings. Similarly, the relatively low physician agreement on economic and sustainability risks likely reflects clinicians’ primary focus on immediate clinical responsibilities, diagnostic safety, treatment effectiveness, and patient communication rather than a lack of ethical sensitivity. Physicians have limited bandwidth to attend to broader sustainability issues, while critical from a policy and global governance perspective, remain peripheral to daily medical decision-making. The FDA encourages the use of an “algorithmic bias evaluation” framework to identify and mitigate discrimination based on race, gender, or geographic region [46,47]; while the United Kingdom’s National Institute for Health and Care Excellence recommends addressing job replacement anxiety through role transformation [48]. In China, primary care institutions lack technology access, training resources, and institutional support, making fairness and professional transition issues more pronounced. This study found that over half of the respondents expressed concerns about diagnostic and treatment inequities caused by flaws in AI algorithms, as well as the digital divide resulting from uneven adoption of AI technologies. Although most physicians were not overly worried about AI threatening their careers, primary care providers reported significantly higher concern than tertiary hospital peers. This disparity reflects the uneven distribution of medical resources and career opportunities across different levels of health care institutions. Therefore, a dual approach is needed—promoting AI integration into primary care to reduce the digital divide, while strengthening ethical governance in tertiary hospitals, clarifying the auxiliary role of AI, and alleviating anxiety.

### Limitations

This study has several limitations. First, the expert sample was relatively small and predominantly clinical, limiting the exploration of legal and digital risk dimensions. Second, convenience sampling overrepresented physicians from tertiary hospitals and senior titles, reducing generalizability to primary care and less-experienced clinicians. Third, the lack of patient perspectives may overlook key ethical risks, such as autonomy, informed consent, and trust. Fourth, the low agreement on economic and sustainability risks may reflect measurement limitations, as items did not explicitly link these risks to physicians’ clinical responsibilities. Future research should diversify expert samples, include patient views, use stratified sampling, and develop more context-sensitive items to assess sustainability risks.

### Conclusion

This study systematically examines the ethical risks of medical AI in China and their impact on physicians’ perceptions. The findings carry several broader implications for policy, governance, and practice. The main conclusions are as follows:

A multidimensional ethical risk framework for medical AI was constructed. Using grounded theory, we constructed a framework with five main categories: (1) physiological risks, (2) psychological risks, (3) data and privacy risks, (4) social risks, as well as (5) economic and sustainability risks—along with 15 subcategories. Despite low physician agreement on economic or sustainability risks, its inclusion is theoretically and ethically justified for sustainable health care. This framework overcomes fragmented existing research and provides a foundation for systematic risk governance.

Physicians’ perceptions and primary concerns regarding the ethical risks of medical AI were revealed. A survey of 600 physicians revealed concentrated concerns, such as data privacy leakage (over 67% expressed a high level of concern), ambiguous accountability (75.7% believed it requires urgent attention), and a physician-patient trust crisis (approximately 70% indicated concern). Thus, data security, accountability, and preserving physician-patient relationships are foremost ethical priorities.

Multiple regression showed that physicians’ familiarity with AI, specialized AI training, and hospital-level AI ethics review procedures significantly affect risk awareness. Specialized training increased sensitivity to data privacy risks and social risks, such as fairness concerns, trust crisis, and ambiguous accountability. Established ethics review protocols positively correlated with awareness of physiological risks, data and privacy risks, and social risks.

In summary, this study enriches the theoretical framework and provides empirical evidence for targeted governance. Future efforts should strengthen AI ethics training for physicians, improve institutional ethics review, clarify accountability systems, and integrate AI into primary care to reduce the digital divide.

## Supplementary material

10.2196/89300 Multimedia Appendix 1 Informed consent form and questionnaire on doctors’ perceptions.

10.2196/89300 Checklist 1 COREQ checklist.

10.2196/89300 Checklist 2 STROBE checklist.

## Abbreviations

AI: artificial intelligence
COREQ: Consolidated Criteria for Reporting Qualitative Research
FDA: US Food and Drug Administration
GDPR: General Data Protection Regulation
HIPAA: Health Insurance Portability and Accountability Act
STROBE: Strengthening the Reporting of Observational studies in Epidemiology
WHO: World Health Organization
