# Supplementary material for: Portraying Ethical Risks of Medical AI: Mixed Methods Study From Connotation Definition to a Survey on Physicians’ Cognition
Source: J Med Internet Res. 2026 Jul 9;28:e89300. doi: 10.2196/89300 (PMC13348801; doi:10.2196/89300)
Supplement: Multimedia Appendix 1 [file jmir-v28-e89300-s001.pdf]

Supplementary File S3

Informed Consent Form for Electronic Consent

Study Title: Portraying Ethical Risks of Medical AI: A Mixed-Methods Study From Connotation Definition to a Survey on Physicians' Cognition

Principal Investigator: Prof. Zhongguang Yu, Respiratory Centre, China-Japan Friendship Hospital

IRB Approval No.: [2024-KY-254] (Ethics Committee of China-Japan Friendship Hospital)

Dear Participant,

You are invited to participate in a study on the willingness of Physicians to adopt artificial intelligence (AI) tools. Please read the following information carefully before deciding whether to participate.

1. Purpose

With the rapid development of artificial intelligence technology in the medical field, it has shown great potential in assisting diagnosis, treatment decision-making, health management, and other aspects, but it has also brought a series of ethical risks (such as privacy breaches, unclear responsibilities, algorithm bias, etc.). At present, doctors' understanding of these ethical risks is not yet clear. This study aims to understand your views on the ethical risks of medical AI and their influencing factors through a questionnaire survey, providing a basis for formulating more reasonable AI ethical governance policies.

2. Procedures

This questionnaire is divided into two parts: the first part collects your basic information (such as gender, age, professional title, hospital level, etc.); The second part asks for your views on medical AI in different dimensions, such as physiological risks, psychological risks, data and privacy risks, social risks, and economic and sustainable risks. The questionnaire consists of approximately 25 questions, rated on a 5-point scale (from 'strongly disagree' to 'strongly agree'), and has a small number of open-ended questions. Estimated completion time: approximately 5 minutes. The questionnaire is filled out anonymously or in encoded form, and does not require your name to be provided.

3. Risks and Discomforts

This study involves only an anonymous questionnaire and does not include any medical intervention or collection of sensitive personal information. The risk is no more than minimal (comparable to routine daily thinking).

4. Benefits

There is no direct financial or medical benefit to participants. However, your participation will help advance the appropriate application of AI in medical, indirectly promoting the quality and efficiency of medical research.

5. Confidentiality

All collected data will be strictly confidential. Data are encrypted and accessible only to core research team members. Results will be published in aggregated form, and no individual responses will be disclosed. Your anonymity is fully protected.

6. Voluntary Participation and Withdrawal

Participation is entirely voluntary. You may refuse to participate or withdraw at any time before submitting the questionnaire without providing any reason, and without any negative impact on your work, position, or benefits. Due to anonymity, once submitted, individual responses cannot be withdrawn or identified.

7. Contact Information

For questions about this study or to request study results, please contact:

Principal Investigator: Prof. Zhongguang Yu

Email: yzg081892@163.com

For ethical concerns or complaints, please contact:Ethics Committee of China-Japan Friendship Hospital

Electronic Consent

By clicking the "I have read and agree to participate" button below, you confirm that:

You have read and understood the above information;

You voluntarily agree to participate in this study;

You agree that the research team may collect and use your anonymous questionnaire data as described in this consent form.

If you do not agree, please close this page without taking any action.

[I have read and agree to participate] ← (button)

Questionnaire on Doctors' Perception and Causes of Ethical Risks in the Clinical Application of Medical AI

Dear friend:

Hello! With the rapid development of artificial intelligence (AI) technology in the medical field, it has greatly changed the work experience of doctors. In order to gain a deeper understanding of the current application status of AI technology in medical scenarios, especially doctors' ethical awareness of medical AI, we conducted this survey.

This questionnaire is divided into two parts. Part 1: Basic information of experts; Part 2: Investigation on Doctors' Perception of Ethical Risks in the Clinical Application of Medical AI; There are 5 measures in the questionnaire, and please rate them according to your willingness: 5="strongly agree", 4="agree", 3="average", 2="disagree", 1="strongly disagree". The questionnaire has an open-ended Q&A section, and you are requested to provide your opinions and suggestions.

This questionnaire will take approximately 5 minutes to complete, and there is no right or wrong answer to any of the questions. Please feel free to answer based on your actual situation. We will strictly keep confidential the information collected from the questionnaire and use it only for academic research. If you have any questions during the filling process, please feel free to contact us at any time. Thank you for taking the time to support our research!

1. Your gender: (1) Male (2) Female

2. Your age: (1) Under 30 years old (2) 30-39 years old (3) 40-49 years old (4) 50 years old and above

3. Your level of education [single choice]

(1) Undergraduate (2) Master's (3) Doctoral (4) Other__________

4.Your professional title：

☐resident physician ☐attending physician ☐Associate Chief Physician ☐Chief Physician

*5. Your department _____

*6. Your years of work experience

☐<5 years ☐5-10 years ☐10-15 years ☐>15 years

*7. Level of your medical institution

☐tertiary hospital ☐secondary hospital ☐Primary healthcare institutions

*8. Annual outpatient volume of the hospital

☐<50000 people ☐50000 to 100000 people ☐100000 to 300000 people ☐>300000 people

*9. Familiarity with clinical applications of medical Al

☐Very unfamiliar ☐unfamiliar ☐General ☐familiar with ☐very familiar

*10. Acceptance of AI specialized training

☐systematic course ☐short-term training ☐self-study ☐Nothing

*11. Have you ever used AI in your clinical diagnosis and treatment process? Yes☐ No☐

*12. Are you willing to use AI in clinical diagnosis and treatment work?

☐very willing ☐willing ☐kind ☐unwilling ☐Very unwilling

*13. AI Scenarios already deployed in hospitals (Multiple Choice)

【 Select at least 1 item, 0 items have been selected 】

☐No deployment ☐prognostic prediction ☐image recognition ☐health management

☐Pathological diagnosis ☐surgical navigation ☐Medication recommendations ☐other

14. Has the hospital established an AI technology ethics review process

☐Standardized processes have been established

☐There is an informal process

☐Nothing

☐not clear

Part 2: Investigation on Ethical Risk Perception of Clinical Application of Medical AI

*1. I am concerned that medical AI may make mistakes in disease diagnosis, causing patients to miss the best treatment opportunity.

☐Strongly disagree ☐disagree ☐general ☐agree ☐Strongly agree

*2. I am concerned that medical AI may make mistakes in disease treatment, leading to poor prognosis for patients and even endangering their lives.

☐Strongly disagree ☐disagree ☐general ☐agree ☐Strongly agree

*3. The clinical application of AI technology has made me worry that some clinical work functions may be taken over by AI, leading to anxiety.

☐Strongly disagree ☐disagree ☐general ☐agree ☐Strongly agree

*4. I think patients may experience psychological pressure due to concerns about the accuracy of AI results.

☐Strongly disagree ☐disagree ☐general ☐agree ☐Strongly agree

*5.I think doctors may experience psychological pressure due to their lack of understanding of the decision-making logic of AI technology.

☐Strongly disagree ☐disagree ☐general ☐agree ☐Strongly agree

*6.I believe that patients may experience feelings of concern due to their lack of understanding of AI's decision-making logic, such as "why is this treatment plan recommended.

☐Strongly disagree ☐disagree ☐general ☐agree ☐Strongly agree

*7. I believe that in the process of medical AI data collection, certain non-standard operations (such as insufficient informed consent) may infringe on patient privacy and harm patient rights.

☐Strongly disagree ☐disagree ☐general ☐agree ☐Strongly agree

*8.I am concerned that there may be security vulnerabilities in the storage and access of medical AI data, which could lead to the leakage of patient privacy and cause unnecessary inconvenience to patients.

☐Strongly disagree ☐disagree ☐general ☐agree ☐Strongly agree

*9.I am concerned that patient data may be used for non-medical commercial activities, thereby infringing on patient rights.

☐Strongly disagree ☐disagree ☐general ☐agree ☐Strongly agree

10. This question is a reliability test for this questionnaire. Please select "strongly agree"

☐Strongly disagree ☐disagree ☐general ☐agree ☐Strongly agree

*11.I am concerned that there may be flaws in medical AI algorithms that could result in unfair medical care due to factors such as patient age, gender, ethnicity, occupation, and severity of the condition.

☐Strongly disagree ☐disagree ☐general ☐agree ☐Strongly agree

*12. I am concerned that the application of medical AI may create a "digital divide" due to differences in technological popularity, which will exacerbate the fairness between developed and underdeveloped regions in terms of medical resources.

☐Strongly disagree ☐disagree ☐general ☐agree ☐Strongly agree

*13.I am concerned that the application of medical AI may have a significant impact on the profession of doctors, leading to many people losing their jobs.

☐Strongly disagree ☐disagree ☐general ☐agree ☐Strongly agree

*14. I am concerned that medical AI applications may reduce emotional communication with patients (such as empathy, comfort, etc.).

☐Strongly disagree ☐disagree ☐general ☐agree ☐Strongly agree

15 I am concerned that there may be uncertainties in the diagnostic accuracy and interpretability of decisions in the clinical application of medical AI, which may lead to patients and their families questioning the professional judgment of * doctors.

☐Strongly disagree ☐disagree ☐general ☐agree ☐Strongly agree

16.I am concerned that the unclear division of responsibilities between AI development teams, hospitals, and doctors may lead to legal disputes after AI misdiagnosis and mistreatment.

☐Strongly disagree ☐disagree ☐general ☐agree ☐Strongly agree

17.I am concerned that with the widespread application of AI in clinical settings, the dominant position of doctors in medical decision-making will be weakened.

☐Strongly disagree ☐disagree ☐general ☐agree ☐Strongly agree

18.I am concerned that the use of medical AI may reduce the autonomy of patients and their families (such as participating in doctor-patient decision-making, expressing treatment needs, etc.).

☐Strongly disagree ☐disagree ☐general ☐agree ☐Strongly agree

*19. I am concerned that the introduction of AI technology may impose additional financial burdens on patients, such as paying extra fees

☐Strongly disagree ☐disagree ☐general ☐agree ☐Strongly agree

*20. I am concerned that the excessive or improper use of medical AI may result in the waste of medical resources.

☐Strongly disagree ☐disagree ☐general ☐agree ☐Strongly agree

21. I am concerned that the waste generated by medical AI equipment and facilities in clinical applications may have an impact on the hospital and surrounding environment.

☐Strongly disagree ☐disagree ☐general ☐agree ☐Strongly agree

22. *I am concerned that the use of medical AI devices may increase energy consumption and operational pressure in hospitals.

☐Strongly disagree ☐disagree ☐general ☐agree ☐Strongly agree
