# Supplementary material for: Portraying Ethical Risks of Medical AI: Mixed Methods Study From Connotation Definition to a Survey on Physicians’ Cognition
Source: J Med Internet Res. 2026 Jul 9;28:e89300. doi: 10.2196/89300 (PMC13348801; doi:10.2196/89300)
Supplement: Checklist 1 [file jmir-v28-e89300-s002.pdf]

Supplementary File S1

COREQ (Consolidated Criteria for Reporting Qualitative Research) Checklist

This checklist is based on the 32-item COREQ guidelines for reporting qualitative research (interviews and focus groups).

Domain 1: Research team and reflexivity

Item / Category	Guide Questions / Description	Page No. / Reported on
**Personal Characteristics**		
1. Interviewer/facilitator	Which author/s conducted the interview or focus group?	Page 1
2. Credentials	What were the researcher's credentials? E.g. PhD, MD.	Page 1
3. Occupation	What was their occupation at the time of the study?	Page 1
4. Gender	Was the researcher male or female?	Page 7
5.Experience and training	What experience or training did the researcher have?	Page 7
**Relationship with participants**		
6.Relationship established	Was a relationship established prior to study commencement?	Page 10
7. Participant knowledge of the interviewer	What did the participants know about the researcher? e.g. personal goals, reasons for doing the research.	Page 9
8. Interviewer characteristics	What characteristics were reported about the interviewer/facilitator? e.g. Bias, assumptions, reasons and interests in the research topic.	Page 15

Domain 2: Study design

Item / Category	Guide Questions / Description	Page No. / Reported on
**Theoretical framework**		
9. Methodological	What methodological orientation was stated to	Page 6,9

orientation and Theory	underpin the study? e.g. grounded theory, discourse analysis, ethnography, phenomenology, content analysis.	
**Participant selection**		
10. Sampling	How were participants selected? e.g. purposive, convenience, consecutive, snowball.	Page 13
11. Method of approach	How were participants approached? e.g. face-to-face, telephone, mail, email.	Page 10
12. Sample size	How many participants were in the study?	Page 10,13
13. Non-participation	How many people refused to participate or dropped out? Reasons?	Page 13
**Setting**		
14. Setting of data collection	Where was the data collected? e.g. home, clinic, workplace, etc.	Page 10
15. Presence of non-participants	Was anyone else present besides the participants and researchers?	Page 10
16. Description of sample	What are the important characteristics of the sample? e.g. demographic data, date.	Page 10,13
**Data collection**		
17. Interview guide	Were questions, prompts, guides provided by the authors? Was it pilot tested?	Page 7
18. Repeat interviews	Were repeat interviews carried out? If yes, how many?	NA
19. Audio/visual recording	Did the research use audio or visual recording to collect the data?	Page 7
20. Field notes	Were field notes made during and/or after the interview or focus group?	Page 7
21. Duration	What was the duration of the interviews or focus group?	Page 7
22. Data saturation	Was data saturation discussed?	Page 7
23. Transcripts returned	Were transcripts returned to participants for comment and/or correction?	Page 10

Domain 3: Analysis and findings

Item / Category	Guide Questions / Description	Page No. / Reported on
**Data analysis**		
24. Number of data coders	How many data coders coded the data?	Page 8
25. Description of the coding tree	Did authors provide a description of the coding tree?	Page 13
26. Derivation of themes	Were themes identified in advance or derived from the data?	Page 7-9
27. Software	What software, if applicable, was used to manage the data?	Page 11
28. Participant checking	Did participants provide feedback on the findings?	Page 9
**Reporting**		
29. Quotations presented	Were participant quotations presented to illustrate the themes/findings? Was each quotation identified? e.g. participant number.	NA
30. Data and findings consistency	Was there consistency between the data presented and the findings?	Page 11
31. Clarity of major themes	Were major themes clearly presented in the findings?	Page 27，28
32. Clarity of minor themes	Is there a description of diverse cases or discussion of minor themes?	Page 23
